# Children exposed to hydroxychloroquine in utero show no signs of retinal toxicity at age 5

**DOI:** 10.1136/lupus-2026-002078

**Published:** 2026-05-27

**Authors:** Peter M Izmirly, Mala Masson, Philip M Carlucci, Nathalie Costedoat-Chalumeau, Noël Zahr, Mimi Y Kim, Jill P Buyon, Michael F Marmor

**Affiliations:** 1 Division of Rheumatology, NYU Grossman School of Medicine, New York, New York, USA; 2 Hospital Cochin, Paris, France; 3 Pitié-Salpêtrière University Hospital, Paris, France; 4 Division of Biostatistics, Department of Epidemiology and Population Health, Albert Einstein College of Medicine, Bronx, New York, USA; 5 Department of Ophthalmology, Stanford University School of Medicine, Stanford, California, USA

**Keywords:** Systemic Lupus Erythematosus, Sjogren's Syndrome, Autoantibodies

## Abstract

**Objective:**

Limited examinations from prior studies support the absence of retinal toxicity in infants exposed to maternal hydroxychloroquine (HCQ). However, the infant retina does not have adult morphology that could show typical signs of HCQ retinopathy. Using the Preventive Approach To Congenital Heart Block with Hydroxychloroquine (PATCH) study, children at age 5 (when adult morphology has been reached) were evaluated using modern imaging techniques.

**Methods:**

Ocular coherence tomography (OCT) was performed (CIRRUS HD-OCT, Carl Zeiss Meditec) at age 5 on 10 PATCH children exposed to maternal HCQ 400 mg daily and 16 healthy controls unexposed to HCQ matched for gestational age at birth, sex and race/ethnicity. Cross-sectional images were examined by a retinal specialist blinded to exposure history. OCT retinal thickness values were displayed in the 9 Early Treatment Diabetic Retinopathy Study (ETDRS) regions by CIRRUS software and compared with available normative data.

**Results:**

Cord blood levels substantiated HCQ exposure. OCT cross sections were normal with no visible thinning diffusely or focally in the ganglion cell layer, photoreceptors, ellipsoid zone or retinal pigment epithelium. ETDRS regional thickness values were less reliable because of poor resolution with some OCT recordings but showed overall thickness (in micrometres) averaging 297.70±10.53 in controls compared with 288.92±13.77 for those in PATCH, p=0.18. Both values fell within normative data for healthy children.

**Conclusions:**

Cross-sectional OCT anatomy in HCQ-exposed children was normal at age 5 with no evidence of toxicity, providing further reassurance that HCQ use during pregnancy does not result in retinal toxicity in the offspring.

**WHAT IS ALREADY KNOWN ON THIS TOPIC**

**WHAT THIS STUDY ADDS**

- Leveraging data from the Preventive Approach to Congenital Heart Block with Hydroxychloroquine (PATCH) study, children at age 5 (when adult morphology has been reached) previously exposed to hydroxychloroquine and healthy matched unexposed controls were evaluated by optical coherence tomography (OCT). Cord blood levels substantiated HCQ exposure of the children. The OCT cross-sections were normal with no visible thinning diffusely or focally. There were no differences between Early Treatment Diabetic Retinopathy Study regions in controls compared with those in PATCH.

**HOW THIS STUDY MIGHT AFFECT RESEARCH, PRACTICE OR POLICY**

- Using sensitive imaging techniques for the first time to evaluate children unequivocally exposed to HCQ in utero showed no evidence of ocular toxicity. These data provide further reassurance that HCQ use during pregnancy does not result in retinal toxicity in the offspring and should relieve concerns for both physicians and patients.

## Introduction

 Antimalarials are a cornerstone of treatment for systemic lupus erythematosus (SLE) given studies showing an association with reduction in flares^1 2^ and protection against organ damage.^2^,^5^ Data suggest that HCQ is safe to continue during pregnancy^2 6^ with no increased frequency of congenital malformations^7^ or infantile retinal abnormalities.^8 9^ Benefits of HCQ use during pregnancy include reduction of flares^10^,^12^ and a decreased risk of neonatal lupus.^13^,^16^ The American College of Rheumatology and EULAR both recommend maintaining HCQ in SLE patients throughout pregnancy.^17 18^

Despite these known advantages, physicians and patients may remain reluctant to initiate or continue HCQ during pregnancy, given the potential of this drug to cause retinopathy in adults and uncertainty regarding potential toxicity to the developing fetal retina. HCQ-mediated damage to the human retina occurs only after many years of accumulated exposure,^19 20^ suggesting that damage from 9 months or less of fetal exposure is unlikely. Hundreds of infants have been examined after maternal HCQ use without any overt abnormality, and many children have been born to HCQ-taking mothers without reported visual abnormalities later in life.^6 8 9^ However, there are no direct data on possible effects to a developing eye. Moreover, no controlled studies have been done using sensitive imaging (optical coherence tomography, OCT) that can reveal early signs of toxicity in adults before visual symptoms.^19 20^ The OCT changes typically are thinning of photoreceptor layers or the ellipsoid zone in characteristic parafoveal or paracentral regions of the retina.

To resolve this lingering concern about in utero toxicity, several elements would provide more definitive assurance. These include maternal and cord blood HCQ levels to unambiguously support fetal exposure and sophisticated OCT evaluations of the child at an age when the retina has acquired adult morphology to determine whether any early anatomical signs of toxicity are present. Accordingly, we leveraged the Preventive Approach To Congenital Heart Block with Hydroxychloroquine (PATCH) study, a prospective trial the primary objective of which was to evaluate whether HCQ reduced the risk of recurrent cardiac neonatal lupus (cardiac-NL).^16^ In this study, the levels of HCQ were measured at each trimester and at delivery from the mothers and from cord blood. Subsequently, HCQ-exposed children and age/race matched healthy controls were evaluated by OCT at age 5, by which time the retina reliably shows adult morphology.^21^

## Methods

The methods of the PATCH study have been previously described.^16^ Women were enrolled if they met the following inclusion criteria: pregnant less than 10 weeks, a previous pregnancy affected by cardiac-NL, and positive for anti-SSA/Ro antibodies at screening. Exclusion criteria included being on >20 mg prednisone or fluorinated steroids at screening. During this study, pregnancies were monitored with weekly echocardiograms from 16 to 26 weeks for PR intervals, advanced block, and/or cardiomyopathy. The primary outcome was second/third degree block in utero or at birth or an isolated cardiomyopathy.^16^

Adherence to the treatment was assessed by whole blood levels of HCQ measured at baseline, second trimester, third trimester, delivery and in cord blood by high-performance liquid chromatography as previously described.^22^ Maternal health status was classified as SLE based on fulfilment of either American College of Rheumatology^23 24^ or Systemic Lupus International Collaborating Clinics classification criteria^25^ as EULAR/ACR criteria^26^ had not yet been established during the PATCH trial; or classified as Sjögren’s disease (SjD) based on fulfilment of American-European Consensus Group’s criteria for probable SS^27^ or definite SS based on American College of Rheumatology/European League Against Rheumatism criteria.^28^ Mothers that were not classified as SS or SLE were diagnosed as undifferentiated autoimmune syndrome or asymptomatic.^16^

OCT was to be performed on all PATCH children at age 5 years when adult retinal morphology has typically been reached^21^ and unexposed controls were matched as closely as possible for sex, race/ethnicity and gestational age at birth. Controls were children unexposed to HCQ in utero, whose mothers had no known autoimmune or rheumatic disease at the time of OCT. Given the timing of the COVID pandemic, enrolment was halted in March/April 2020. At that time 10 HCQ-exposed children in PATCH and 16 non-HCQ-exposed matched controls had undergone an OCT.

Retinal thickness values were displayed from the nine Early Treatment Diabetic Retinopathy Study (ETDRS) regions by CIRRUS HD-OCT software, Carl Zeiss Meditec machine. Two HCQ-exposed and two unexposed had qualitative data only and thus were not included in the ETDRS analysis. All cross-sectional images were examined by an experienced retina specialist, MFM, with no knowledge of exposure history.^19 20^ Comparisons were made between the HCQ exposed and unexposed children for all nine of ETDRS thickness regions and overall thickness using a Mann-Whitney U test in the RStudio V.2023.12.0.369 ‘tableone’ package. P values were adjusted for multiple comparisons using the Benjamini-Hochberg false discovery rate method. In addition, the HCQ thickness values of exposed children were compared with normative data published after the study was designed.^29 30^

The study was approved by the Institutional Review Board at the NYU Grossman School of Medicine, and the parents provided written informed consent. Neither patients nor the public were involved in the design of the study.

## Results

The demographics of the 10 HCQ exposed and 16 non-HCQ exposed children who underwent an OCT are displayed in table 1.

**Table 1:** Demographics, diagnoses and medications of study participants and controls

Variables	Hydroxychloroquine (HCQ) (n=10)	No HCQ (n=16)
Children characteristics		
Gestational age at delivery (weeks) (mean±SD)	37.5±2.2 weeks	37.7±2.2 weeks
Sex (% F/M)	60/40	62.5/37.5
Child race/ethnicity, N (%)		
White	8 (80)	13 (81)
Black	0 (0)	0 (0)
Hispanic	1 (10)	0 (0)
Asian	0 (0)	3 (19)
Other	1 (10)	0 (0)
Child outcome, N (%)		
Cardiac-neonatal lupus	1 (10)	0 (0)
Cutaneous-neonatal lupus	2 (20)	0 (0)
Abnormal liver function tests	1 (10)	0 (0)
Child age at ophthalmological evaluation (mean±SD, years)	5.0±0.3	5.0±0.5

Maternal diagnosis at delivery, N (%)	HCQ (n=9*)	
Asymptomatic/undifferentiated autoimmune syndrome	5 (56)	–
Sjögren’s disease (SjD)	0 (0)	–
Systemic lupus erythematosus (SLE)	0 (0)	–
SLE/SjD	4 (44)	–

Antibody specificities, N (% positive)		
Anti-Ro60	9 (100)	–
Anti-Ro52	9 (100)	–
Anti-La48	7 (78)	–

In utero medication/therapy, N (% exposed)	HCQ (n=10)	
Fluorinated steroids	1 (10)	–
Non-fluorinated steroids	0 (0)	–
Intravenous immunoglobulin	2 (20)	–

* Includes one set of twins.

Five of the PATCH mothers were asymptomatic or had an undifferentiated autoimmune syndrome, and four were diagnosed with SLE and overlapping SjD. All mothers had anti-Ro60 and anti-Ro52 antibodies, and 78% had anti-La antibodies. One of the 10 children had cardiac-NL and three were exposed in utero to additional medication after enrolment, including fluorinated steroids in one and intravenous immunoglobulin (IVIG) in two. Table 2 shows the average and range of HCQ levels at each trimester of pregnancy, at delivery and in the cord blood which confirm in utero exposure.

**Table 2:** Hydroxychloroquine (HCQ) levels during pregnancy and at delivery

Time point	Mean HCQ level (ng/ml)	Range (ng/mL)
First trimester (n=8)	796	50-1270
Second trimester (n=10)	941	487-1445
Third trimester (n=7)	1001	471-1601
Delivery (n=9)	835	50-1661
Cord blood (n=9)	649	114-1349

**Figure 1:**
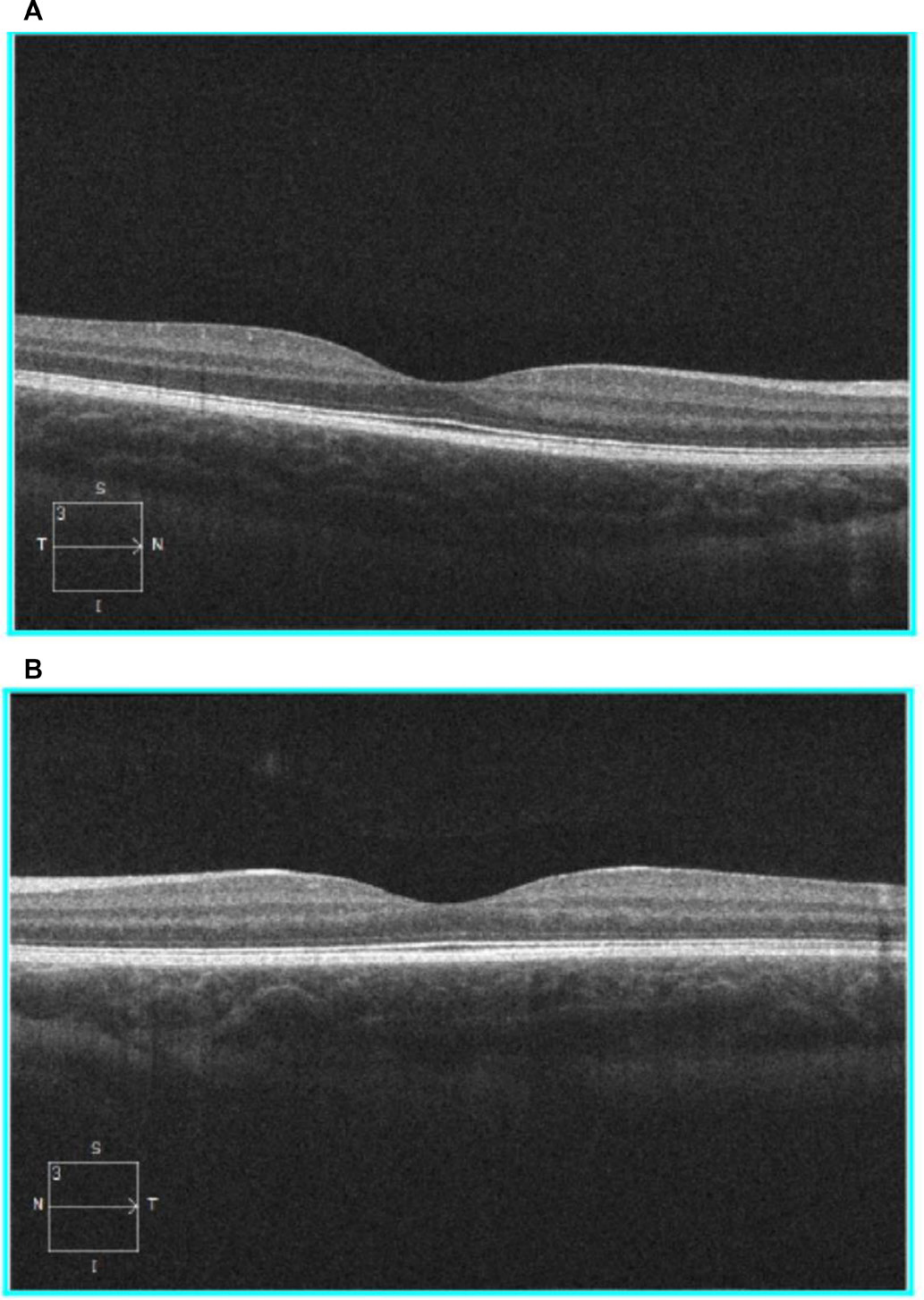
High resolution OCT records from an HCQ-exposed child. (A) Right eye, (B) left eye. HCQ, hydroxychloroquine; OCT, ocular coherence tomography.

All OCT cross-sectional images were generated by different machines, and six OCTs gave low-resolution OCTs, while the remaining had high-resolution images. Nonetheless, all images were carefully examined and confirmed to show clear differentiation of the retinal layers. There were no differences in retinal morphology between HCQ cases and controls with respect to diffuse or any focal thinning of the ganglion cell layer, photoreceptors (outer nuclear) layer, or retinal pigment epithelium, including the parafoveal regions that would be most at risk in white adults.^19 20^ There was no suggestion of thinning at the OCT edges to suggest a pericentral pattern of HCQ damage which has been seen in otherwise healthy Asian individuals.^31^ The high-resolution images displayed well-defined ellipsoid zone lines without disruption. Figure 1 shows a representative high-resolution OCT from a child in the PATCH study.

ETDRS thickness values were available from 8 of the 10 HCQ cases and 14 of the 16 controls (online supplemental table 1A,B, respectively). Of the eight HCQ-exposed patients, two had some regions of interest discarded as artifactual either by the machine or by MFM (online supplemental table 1A). Of the 14 controls, 7 had several regions of interest discarded by the machine (online supplemental table 1B). The acceptable scans showed typical thickness variability in both control and HCQ-exposed groups, with no major outliers among eyes with satisfactory scans. The overall thickness averaged 297.70±10.53 µm in controls compared with 288.92±13.77 µm in HCQ cases, p=0.18. Both values fell within currently available normative data, 279 (95% CI 260 to 298) μm.^29^ The average retinal thickness in the nine ETDRS regions was roughly 10 µm less in HCQ-exposed patients than controls, although these differences were within the SD and the range of values overlapped broadly between both groups. Thus, these differences were deemed not to be clinically significant, and after adjusting for multiple comparisons, none were statistically significant (online supplemental table 1).

## Discussion

Overall, these data are reassuring since the OCT cross-sectional anatomy was normal and similar between HCQ-exposed and unexposed eyes. There was no evidence of either inner or outer retinal thinning or damage, across the macula or regionally (including the parafovea and pericentral retina), in either group of children. The average retinal thickness was slightly lower in those exposed to HCQ, but these differences were within statistical variability for a sample of this size, and the range of values for HCQ and control cases overlapped and were not significantly different after adjusting for multiple comparisons.

Ocular toxicity in infants exposed to antimalarials has been previously reported in several reviews and series^6 8 9^ based on clinical exam and, in some cases, on electrophysiological recordings. Only a small number of older children or adults were examined by viewing the retina and using subjective tests such as visual acuity. None of these data show consistent HCQ toxicity. However, the infant retina is difficult to examine, and electrophysiology is generally unreliable in infants except in the presence of very severe damage. Thus, subtle damage might have been missed. Current evaluation of clinical toxicity in older children and adults can detect damage through the use of sensitive anatomic imaging (OCT), long before visual acuity is affected. The initial OCT changes in toxicity are retinal thinning or damage in off-centre regions that have been found to be most characteristic of HCQ retinopathy.^19 20^ This study is the first to use OCT to critically examine the retina anatomically in children old enough to show adult retinal morphology. The cause of mild foveal thinning in the HCQ subjects prior to adjusting for multiple comparisons is unclear, but probably unrelated to HCQ toxicity. The fovea is never involved in retinopathy until very late and severe stages, and it is not considered a region of interest for screening.

This study has several strengths. The OCT exams were evaluated by one expert ophthalmologist,^19 20^ MFM, blinded to HCQ exposure. By using 5-year-old subjects, the well-known difficulties of assessing infantile vision and retinal normality were avoided, and established adult morphological criteria of HCQ retinopathy could be used. Non-adherence to HCQ among SLE patients has been well documented,^32^ but compliance in PATCH was assured by measuring maternal levels at each trimester and in cord blood. Although it is acknowledged that some cord blood levels were below 500 ng/mL, suggesting minimal exposure, levels in the mother were often higher earlier in pregnancy and thus total exposure in utero may be greater than reflected in the cord blood alone.

The greatest weakness is the limited number of children evaluated, which was unavoidable given the timing of the pandemic relative to when many of the children reached the age window for ocular assessments. Regarding the unexposed controls, we chose not to include anti-SSA/Ro-exposed in the absence of maternal use of HCQ as there was no a priori reason to suspect that these maternal antibodies would result in retinal toxicity.

In sum, data acquired via OCT in children aged 5 whose mothers participated in PATCH provide further reassurance regarding the safety of HCQ use during pregnancy by demonstrating the absence of any anatomical signs of HCQ retinopathy.

## Supplementary material

10.1136/lupus-2026-002078 online supplemental table 1

## Data Availability

All data relevant to the study are included in the article or uploaded as supplementary information.
