# Supplementary material for: Children exposed to hydroxychloroquine in utero show no signs of retinal toxicity at age 5
Source: Lupus Sci Med. 2026 May 27;13(1):e002078. doi: 10.1136/lupus-2026-002078 (PMC13218190; doi:10.1136/lupus-2026-002078)
Supplement: online supplemental table 1 [file lupus-13-1-s001.pdf]

Supplemental Table 1: ETDRS Thickness Values

A. HCQ Exposed

**Patient ID**	Fovea OS	Superior Parafovea OS	Temporal Parafovea OS	Nasal Parafovea OS	Inferior Parafovea OS	Superior Perifovea OS	Temporal Perifovea OS	Nasal Perifovea OS	Inferior Perifovea OS	Fovea OD	Superior Parafovea OD	Temporal Parafovea OD	Nasal Parafovea OD	Inferior Parafovea OD	Superior Perifovea OD	Temporal Perifovea OD	Nasal Perifovea OD	Inferior Perifovea OD
2477	240	285	272	310	*	288	**N/A**	298	**N/A**	216	296	297	312	*	351	263	304	266
2221	234	322	302	316	316	291	260	306	277	237	318	307	312	313	282	264	302	274
2280	235	320	305	312	317	290	305	312	286	234	320	307	312	319	289	275	319	291
2471	243	295	280	295	285	266	249	281	256	274	299	288	299	277	278	259	284	274
2740	290	336	310	333	328	294	290	299	290	271	324	309	330	**N/A**	292	272	296	*
2794	207	286	273	284	286	255	275	239	250	207	289	277	287	287	257	237	274	247
2787	221	302	286	296	295	265	243	282	254	223	304	289	299	296	311	245	285	255
2795	227	327	309	318	319	299	275	313	277	232	325	312	315	324	292	275	309	283
Mean	237.12	309.12	292.12	308.00	306.57	281.00	271.00	291.25	270.00	236.75	309.38	298.25	308.25	302.67	294.00	261.25	296.62	270.00
SD	24.27	19.61	16.14	15.57	17.48	16.39	22.13	24.34	16.36	24.19	14.02	12.54	13.00	18.85	27.62	13.95	14.85	15.34
Median	234.50	311.00	294.00	311.00	316.00	289.00	275.00	298.50	277.00	233.00	311.00	302.00	312.00	304.50	290.50	263.50	299.00	274.00
IQR	225.50 - 240.75	292.75 - 323.25	278.25 - 306.00	295.75 - 316.50	290.50 - 318.00	265.75 - 291.75	254.50 - 282.50	281.75 - 307.50	255.00 - 281.50	221.25 - 245.50	298.25 - 321.00	288.75 - 307.50	299.00 - 312.75	289.25 - 317.50	281.00 - 296.75	255.50 - 272.75	284.75 - 305.25	260.50 - 278.50

B. Unexposed Healthy Controls

**Patient ID**	Fovea OS	Superior Parafovea OS	Temporal Parafovea OS	Nasal Parafovea OS	Inferior Parafovea OS	Superior Perifovea OS	Temporal Perifovea OS	Nasal Perifovea OS	Inferior Perifovea OS	Fovea OD	Superior Parafovea OD	Temporal Parafovea OD	Nasal Parafovea OD	Inferior Parafovea OD	Superior Perifovea OD	Temporal Perifovea OD	Nasal Perifovea OD	Inferior Perifovea OD
9904	254	315	300	316	307	288	267	306	273	261	325	309	325	322	292	270	305	286
9905	281	321	302	325	315	320	268	307	**N/A**	268	319	298	324	311	283	260	299	278
9906	247	321	298	320	311	282	269	305	283	252	323	301	322	313	300	263	312	283
9907	272	328	312	327	321	290	271	306	281	283	346	328	348	337	306	282	329	294
9908	288	323	307	333	321	283	268	305	277	279	325	304	336	320	295	268	318	290
9909	264	347	341	337	**N/A**	289	291	294	271	298	339	341	343	345	312	298	308	287
9910	258	308	306	300	284	278	272	302	277	257	**N/A**	314	330	268	**N/A**	300	**N/A**	297
9911	250	335	322	333	320	299	291	**N/A**	291	244	330	317	325	324	304	286	314	285
9912	267	330	320	331	328	300	280	304	272	260	296	315	325	305	316	274	299	258
9913	260	300	293	303	284	305	252	280	**N/A**	252	309	304	310	252	**N/A**	253	275	220
9914	279	337	326	331	329	287	283	316	279	273	325	305	320	318	298	261	310	283
9915	221	308	279	302	**N/A**	279	**N/A**	303	**N/A**	224	262	285	301	287	302	**N/A**	309	**N/A**
9916	219	303	302	307	316	270	263	293	265	218	274	296	302	309	296	261	290	268
9917	284	325	312	318	306	**N/A**	278	298	309	237	246	271	296	299	250	**N/A**	289	274
Mean	260.29	321.50	308.57	320.21	311.83	290.00	273.31	301.46	279.82	257.57	309.15	306.29	321.93	307.86	296.17	273.00	304.38	277.15
SD	21.27	13.63	15.41	12.84	14.85	13.22	11.06	8.74	11.87	22.30	30.75	17.20	15.37	25.13	17.00	15.36	14.06	20.16
Median	262.00	322.00	306.50	322.50	315.50	288.00	271.00	304.00	277.00	258.50	323.00	304.50	324.50	312.00	299.00	269.00	308.00	283.00
IQR	251.00 - 277.25	309.75 - 329.50	300.50 - 318.00	309.25 - 331.00	306.75 - 321.00	282.00 - 299.00	268.00 - 280.00	298.00 - 306.00	272.50 - 282.00	246.00 - 271.75	296.00 - 325.00	298.75 - 314.75	312.50 - 328.75	300.50 - 321.50	294.25 - 304.50	261.00 - 283.00	299.00 - 312.00	274.00 - 287.00
p-value	0.04	0.13	0.07	0.08	0.67	0.49	0.72	0.54	0.34	0.06	0.42	0.32	0.06	0.59	0.22	0.26	0.21	0.16
q-value	0.29	0.39	0.29	0.29	0.71	0.63	0.72	0.65	0.51	0.29	0.58	0.51	0.29	0.66	0.44	0.47	0.44	0.41

N/A= value discarded by the machine, * value discarded by MFM as artifactual, OS=Left eye, OD right eye, SD = Standard deviation, IQR = interquartile range. Statistics calculated using RStudio version 2023.12.0.369 'tableone' package. P-values from Mann-Whitney U tests were adjusted for multiple comparisons using the Benjamini-Hochberg false discovery rate method and reported as q-values.
